# The frequency and timing of leukopenia and CMV infections in kidney transplant recipients in Finland – a retrospective observational study

**DOI:** 10.3389/ti.2026.16893

**Published:** 2026-10-01

**Authors:** Ilkka Helanterä, Fernanda Ortiz, Essi Havula, Samuli Tuominen, Markus Hakamäki, Tapio Hellman, Satu Mäkelä, Riku Korhonen

**Affiliations:** 1 Transplantation and Liver Surgery, Helsinki University Hospital, University of Helsinki, Helsinki, Finland; 2 Helsinki University Hospital, Abdominal Center, Nephrology, Helsinki, Finland; 3 Medaffcon Oy, Espoo, Finland; 4 Kidney Center, Turku University Hospital and University of Turku, Turku, Finland; 5 Department of Internal Medicine, Tampere University Hospital, Tampere, Finland; 6 Faculty of Medicine and Health Technology, Tampere University, Tampere, Finland; 7 MSD Finland, Espoo, Finland

**Keywords:** CMV, kidney transplantation, leukopenia, neutropenia, valganciclovir

## Abstract

This retrospective observational study investigated the frequency of post-transplant leukopenia and neutropenia (PTLN) and its association with cytomegalovirus (CMV) infections, clinical outcomes, and healthcare resource utilization (HCRU) in kidney transplant recipients (KTRs) in Finland. Clinical and HCRU data were collected from electronic medical records and national registries for the years 2013–2022. Clinical outcomes were assessed during the first 18 months, and cost-related outcomes were evaluated up to 4 years post-transplant. The cohort included 966 KTRs, with the following donor/recipient CMV serostatus distribution: R+ 706 (73.1%), D+/R─ 200 (20.7%), and D─/R─ 60 (6.2%). At 12 months, PTLN occurred in 224 (31.7%) R+, 103 (51.5%) D+/R─, and 6 (10.0%) D─/R─ recipients (p < 0.001). Valganciclovir prophylaxis was started in 28.0% of R+ and 95.0% of D+/R─ patients. CMV DNAemia occurred in 357 (50.6%) R+ and 106 (53.0%) D+/R─ recipients, with median time to CMV DNAemia of 66 days (IQI: 48–110) in R+ and 239 days (IQI: 175–323) in D+/R─ recipients (p < 0.001). PTLN and D+/R─ serostatus were associated with higher HCRU and increased healthcare costs. In conclusion, PTLN was more common in CMV D+/R─ KTRs and both D+/R─ serostatus and PTLN were linked to greater healthcare utilization and cost burden.

## Graphical Abstract

**Figure:**
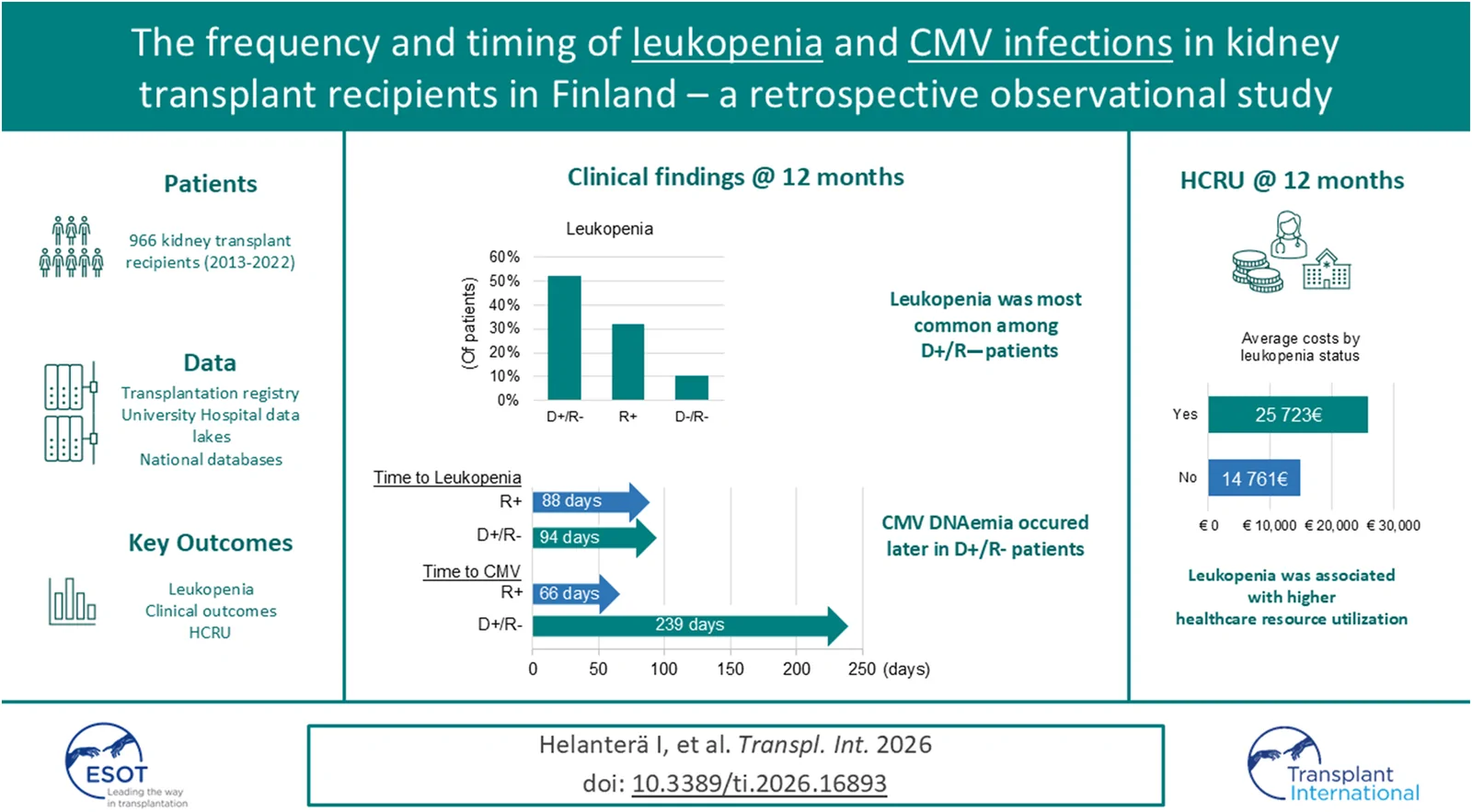

## Introduction

Post-transplant leukopenia or neutropenia (PTLN) is a common complication among kidney transplant recipients (KTRs). Within the first year after transplantation, leukopenia and neutropenia have been reported in 19%–83% and 13%–48% of KTRs, respectively [1]. Risk factors for PTLN include seropositive donor/seronegative recipient (D+/R−) CMV serostatus, as well as myelosuppressive medications such as valganciclovir, ganciclovir, mycophenolate mofetil (MMF), tacrolimus, and sulphamethoxazole/trimethoprim, all of which are commonly used in KTRs [1, 2]. The incidence and associated clinical outcomes of PTLN in KTRs are still partially characterized. PTLN is associated with an increased risk of post-transplant infections including cytomegalovirus (CMV), higher hospitalization rate, acute rejection, graft loss, and mortality [3, 4].

CMV infection is the most frequent infectious complication after solid organ transplantation (SOT) [5] and it contributes to the development of leukopenia or neutropenia in KTRs [6]. Without prophylaxis, symptomatic CMV infection occurs in approximately 10%–20% of KTRs [7]. The prevention and management of CMV infections are critical as CMV is associated with reduced kidney function, increased incidence of acute rejections, and higher mortality among KTRs [5, 8–10].

KTRs with D+/R-status are at the highest risk of CMV infection [11]. In Finland, CMV seroprevalence is estimated at 70%, and approximately 20% of Finnish KTRs fall into the high-risk D+/R─ group [12–15]. Since 2004, D+/R─ KTRs in Finland have received a 6-month valganciclovir prophylaxis. Approximately 40% of these patients acquire primary CMV infection [16, 17]. R+ patients are routinely monitored for CMV DNAemia after transplantation and in case of any symptoms attributable to CMV. A 3-month valganciclovir prophylaxis is used in patients receiving ABO-incompatible transplants or lymphocyte-depleting induction, and after treatment of acute rejection.

While PTLN has been associated with adverse outcomes [1], the economic impact has been characterized in less detail. This study utilized Finnish healthcare registries and three hospital data lakes, covering approximately 50% of the Finnish population, to study the occurrence of PTLN and CMV infections, and healthcare resource utilization (HCRU) among Finnish KTRs between 2013 and 2022.

## Materials and methods

### Data collection

This non-interventional, retrospective registry study used data collected from KTRs, whose (first) transplantation occurred during 2013–2022 and who were from the Helsinki and Uusimaa, Pirkanmaa or Varsinais-Suomi wellbeing counties (combined population approximately 2 million). Patients with other organ transplants (except skin grafts) prior to the kidney transplant were excluded. KTRs were identified from the Finnish Kidney Transplant Registry (FKTR) covering all KTs in Finland. Patient data were collected from the FKTR, the regional Helsinki and Uusimaa, Pirkanmaa, and Varsinais-Suomi wellbeing county data lakes, the national registers of specialty care (HILMO), Social Insurance Institute (SII), and Statistics Finland (SF). The data from FKTR included transplant-related data, recipient and donor characteristics, and post-transplant outcome data. Data from the wellbeing county data lakes included all specialty care diagnoses, hospital-based medications (prescriptions and administrations), and laboratory findings. Data from HILMO included all inpatient and outpatient contacts to specialty healthcare. Data from SII included all reimbursed pharmacy-based medication purchases. Times of death were obtained from SF.

Data collection used unique personal identity codes universally used in the Finnish healthcare. Findata, the Finnish Social and Health Data Permit Authority, pseudonymized all patient identifiers before release of the data into a secure analysis environment. Only anonymous data were exported out of the secure data analysis environment for reporting. All numbers of patients between 1 and 2 were replaced with “<3” to prevent identification of individuals according to the Findata guidelines for secondary use of health and social data [18].

### Outcomes

PTLN was defined based on the laboratory findings with leukocyte count below 3.4 × 10^9^ cells/l and neutrophil count below 1.0 × 10^9^ cells/l. The occurrence of PTLN was defined as the date of the first leukopenia or neutropenia. D─/R─ KTRs due to low PTLN incidence and patients whose follow-up was less than 18 months were not included in landmark analyses according to PTLN status, to focus on the early post-transplant period (Supplementary Material). Immunosuppressive regimens are further described in Supplementary Material.

CMV DNAemia was monitored in the peripheral blood by nucleic acid testing in plasma, with a test result >35 IU/mL considered positive (Supplementary Material). Clinically significant CMV DNAemia and high CMV DNAemia were defined as ≥1 000 IU/mL and ≥10,000 IU/mL, respectively. The onset of a PTLN or CMV DNAemia episode was defined as the first positive laboratory finding, and the episode was considered protracted if additional positive results occurred within 4 weeks of the preceding positive finding. If there was a gap longer than 4 weeks or a negative result, the PTLN or CMV DNAemia episode was considered ended at the latest positive finding. The beginning of a new episode was observed if a new positive CMV nucleic acid test result was detected. Dates of the first, second, third, and fourth PTLN and CMV DNAemia were collected and used as the starting dates of episodes when assessing the timing and order of PTLN and CMV DNAemia in multistate models.

Patient survival was defined as the time from KT until death (all-cause; event). Graft survival was defined as the time from KT to the date of return to dialysis or re-transplantation (event). All patients without events were censored at end of follow-up which was set to December 31st, 2023.

### Healthcare resource utilization

The direct costs were estimated by linking publicly available standard unit costs [19] to inpatient and outpatient visits (by contact type and specialty). The unit costs include the average cost of procedures, operations, laboratory examinations, inpatient medications, and overheads related to the visit. The unit costs were inflation corrected to 2025 level using the price index of public healthcare expenditure. The costs for the medication purchases used the most recent publicly available list price (2024). For the HCRU analysis, costs from the first 30 days after kidney transplantation were excluded to minimise the influence of expected transplantation-related, perioperative, and immediate postoperative costs, thereby focusing on healthcare utilisation after the initial transplant period.

### Statistical analysis

For categorial variables, numbers and proportion of patients per category are reported while for continuous variables the mean and standard deviation (SD) or median and inter-quantile interval (IQI) are reported. Categorial variables were tested with Chi-squared test or Fisher’s exact test if any cell had the number of patients between 1 and 4. If any cell had 0 patients, the test was not performed. Means and medians of continuous variables were tested with a Student’s t-test or a Mann-Whitney U test, respectively. Patient and graft survival were estimated with the Kaplan-Meier method while cumulative incidences of (recurrent) neutropenia, leukopenia, PTLN, and CMV DNAemia, and related composite events were estimated using the Aalen-Johansen method, where death was considered a competing risk. The association of PTLN was assessed with multivariable logistic regression, including recipient and donor demographics, primary kidney disease, delayed graft function, induction therapy, CMV serostatus, valganciclovir use, calcineurin inhibitor use, Charlson comorbidity index (CCI), and panel reactive antibody (PRA) status as covariates. Dialysis type/pre-emptive transplantation status had no impact on the model and was left out from the final analysis. The association of patient and graft survival with the PTLN status were assessed with landmark analyses and using both the Kaplan-Meier method and multivariable Cox proportional hazards (PH) model while adjusting for the same covariates as previously listed. Body mass index (BMI) was not included in the model due to high rate of missing values. The PH assumption was checked visually with Schoenfeld residuals. Costs related to HCRU were analyzed with the mean cumulative functions. Furthermore, a multivariable gamma regression model with log link and the log of the patient-wise follow-up length as offset was fit to assess the association of CMV serotype, PTLN status, and important covariates with the total HCRU related costs 4 years post-KT. Age at transplant and donor age were centered to their mean values. Mean ratios with 95% confidence intervals (CIs) and p-values are reported.

All analyses and data processing were performed using R, a language and environment for statistical computing and graphics [20], version 4.4.1. All p-values below 0.05 were considered statistically significant and where applicable 95% CIs were reported. No adjustment for multiple testing was applied. The number and proportion of missing values are reported where applicable.

## Results

### Patient population

In total, 966 KTRs met the inclusion criteria of the study (Figure 1). Of these, 706 (73.1%) KTRs were in the D+ or D─/R+ (later “R+”), 200 (20.7%) in the D+/R─, and 60 (6.2%) in the D─/R─ CMV serotype subgroups (Table 1). 90.4% of the grafts were from a deceased donor, and the median donor age was 58 years. 69.0% of KT patients did not receive induction therapy; 18.4% received basiliximab and 12.5% ATG (Table 2). 95% of the D+/R─ patients started valganciclovir prophylaxis. Of R + KTRs, 28.0% received valganciclovir prophylaxis and 72.0% were monitored for CMV DNAemia (Table 2). Delayed graft function (DGF) was reported in 19.5% of patients.

**FIGURE 1:**
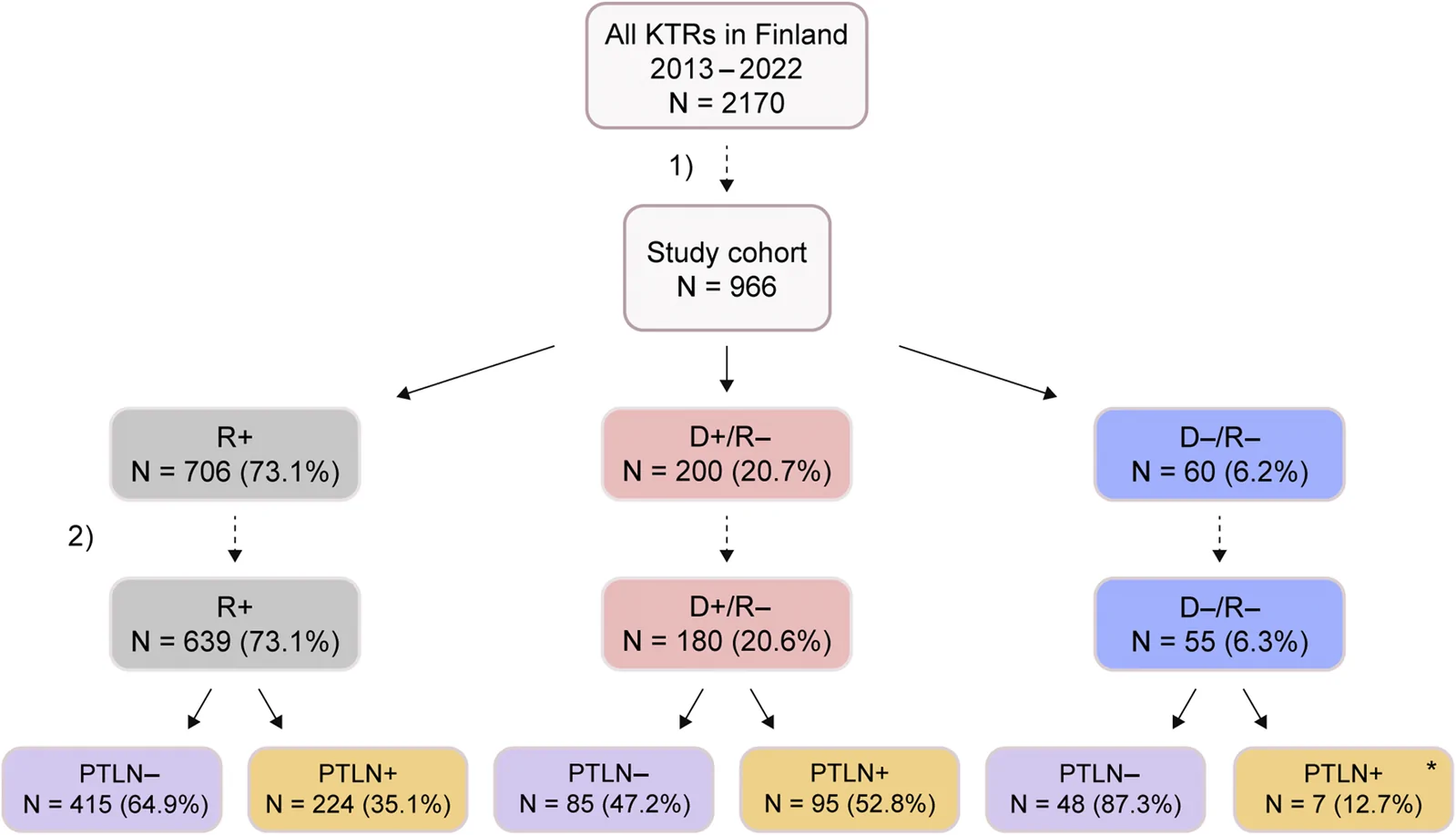
Cohort formation. Abbreviations: D: donor, KTR: kidney transplant recipient, PTLN: post-transplant leukopenia or neutropenia, R: recipient. 1) Inclusion criteria: home municipality Helsinki and Uusimaa, Pirkanmaa, or Varsinais-Suomi, ≥18 years of age at KT, functional graft, no records of letermovir post KT, and CMV serotype at KT available. 2) For landmark analyses, including transplantation outcomes and mortality according to PTLN status, the patients who were not alive at 18 months of follow-up or whose follow-up didn’t extend beyond 18 months were excluded. *) due to the low number of PTLN+, D-/R- KTRs were excluded from the landmark analyses.

**TABLE 1:** Characteristics.

Baseline variable	​	All	R+	D+/R─	D─/R─	P-value
		N = 966	N = 706	N = 200	N = 60	
		​	−73.10%	−20.70%	−6.20%	
Age at KTP, years, median (IQI)		55 (44, 64)	56 (45, 65)	54 (42, 63)	46 (34, 60)	<0.001
Recipient sex	Male	605 (62.6%)	426 (60.3%)	135 (67.5%)	44 (73.3%)	0.038
	Female	361 (37.4%)	280 (39.7%)	65 (32.5%)	16 (26.7%)	
Primary kidney disease	Glomerulonephritis ^1^	246 (25.5%)	187 (26.5%)	37 (18.5%)	22 (36.7%)	0.007
	Diabetes mellitus ^2^	264 (27.3%)	181 (25.6%)	70 (35.0%)	13 (21.7%)	
	PKD ^3^	192 (19.9%)	150 (21.2%)	35 (17.5%)	7 (11.7%)	
	Other	138 (14.3%)	91 (12.9%)	35 (17.5%)	12 (20.0%)	
	Unknown ^4^	126 (13.0%)	97 (13.7%)	23 (11.5%)	6 (10.0%)	
KT year	2013	78 (8.1%)	56 (7.9%)	14 (7.0%)	8 (13.3%)	0.5
	2014–2015	90 (9.3%)	67 (9.5%)	20 (10.0%)	3 (5.0%)	
	2016–2017	278 (28.8%)	193 (27.3%)	67 (33.5%)	18 (30.0%)	
	2018–2019	202 (20.9%)	150 (21.2%)	39 (19.5%)	13 (21.7%)	
	2020–2022	318 (32.9%)	240 (34.0%)	60 (30.0%)	18 (30.0%)	
CCI	0–1	502 (52.0%)	371 (52.5%)	99 (49.5%)	32 (53.3%)	0.01
	2	263 (27.2%)	173 (24.5%)	70 (35.0%)	20 (33.3%)	
	3+	201 (20.8%)	162 (22.9%)	31 (15.5%)	8 (13.3%)	
Donor age, years, median (IQI)		58 (46, 66)	58 (47, 66)	57 (45, 68)	50 (37, 63)	0.004
Donor type	Living	93 (9.6%)	62 (8.8%)	21 (10.5%)	10 (16.7%)	0.12
	Deceased	873 (90.4%)	644 (91.2%)	179 (89.5%)	50 (83.3%)	
ABOi		17 (1.8%)	13 (1.8%)	Censored	<3	0.9
HLA A-, B-, and DR- mismatch, mean (SD)		3.02 (1.29)	3.01 (1.28)	3.09 (1.29)	2.88 (1.35)	0.5
Cold ischemia time, hours, median (IQI)		14 (9, 18)	14 (9, 18)	13 (8, 17)	14 (8, 19)	0.2
Graft function type	EF	778 (80.5%)	565 (80.0%)	169 (84.5%)	44 (73.3%)	0.13
	DGF	188 (19.5%)	141 (20.0%)	31 (15.5%)	16 (26.7%)	
Induction type	None	667 (69.0%)	487 (69.0%)	138 (69.0%)	42 (70.0%)	0.8
	ATG	121 (12.5%)	86 (12.2%)	29 (14.5%)	6 (10.0%)	
	Basiliximab	178 (18.4%)	133 (18.8%)	33 (16.5%)	12 (20.0%)	

Abbreviations: CCI: charlson comorbidity index, DGF: delayed graft function, D: donor, EF: early function, IQI: inter-quartile interval, PKD: polycystic kidney disease, R: recipient, SD: standard deviation.

1 ICD10: N00, N01, N03-N08.

2 ICD10: E10, E11, N08.32.

3 ICD10: Q61, excl. Q61.5.

4 ICD10: N18.

**TABLE 2:** Outcomes.

Outcome		All N = 966	R+ N = 706	D+/R− N = 200	D-/R− N = 60	P-value	R+ PTLN−N = 415	R+ PTLN+N = 224	P-value	D+/R─ PTLN−N = 85	D+/R─ PTLN+N = 95	P-value
PTLN rate at 12 mo*		34.5 (31.7, 37.7)	31.7 (28.5, 35.4)	51.5 (45.0, 58.9)	10.0 (4.7, 21.4)	<0.001	-	-	-	-	-	-
PTLN+ within 18 months of KT, yearly	2013	22 (2.3%)	14 (2.0%)	censored	<3	<0.001	-	-	-	-	-	-
	2014–2015	35 (3.6%)	27 (3.8%)	8 (4.0%)	0	​	-	-	-	-	-	-
	2016–2017	106 (11.0%)	73 (10.3%)	29 (14.5%)	4 (6.7%)	​	-	-	-	-	-	-
	2018–2019	80 (8.3%)	58 (8.2%)	censored	<3	​	-	-	-	-	-	-
	2020–2022	83 (8.6%)	52 (7.4%)	censored	<3	​	-	-	-	-	-	-
Leukopenia rate at 12 mo*		34.5 (31.7, 37.7)	31.7 (28.5, 35.4)	51.5 (45.0, 58.9)	10.0 (4.7, 21.4)	<0.001	-	-	-	-	-	-
Neutropenia rate at 12 mo*		15.1 (13.0, 17.5)	13.6 (11.3, 16.4)	24.0 (18.8, 30.7)	3.3 (0, 13.0)	<0.001	-	-	-	-	-	-
End of follow-up before 18 months		92 (9.5%)	5 (8.3%)	67 (9.5%)	20 (10.0%)	0.81	-	-	-	-	-	-
Time until PTLN [among PTLN + only; months; median (IQI)]		3.0 (1.8, 5.3)	2.9 (1.5, 4.9)	3.1 (2.2, 5.4)	8.7 (4.4, 10.8)	0.032	-	2.9 (1.5, 4.9)	-	-	3.06 (2.17, 5.42)	-
​	Missing (no PTLN)	640 (66.3%)	482 (68.3%)	105 (52.5%)	53 (88.3%)	​	415 (100.0%)	0 (0.0%)	​	85 (100.0%)	0 (0.0%)	​
CMV DNAemia during follow-up		468 (48.8%)	357 (50.6%)	106 (53.0%)	5 (8.3%)	<0.001	196 (47.2%)	121 (54.0%)	0.1	39 (45.9%)	57 (60.0%)	0.058
CMV DNAemia rate at 12 mo*		44.3 (41.2, 47.5)	47.2 (43.6, 51.0)	45.0 (38.6, 52.5)	6.7 (2.6, 17.2)	<0.001	43.1 (38.6, 48.2)	50.9 (44.7, 57.9)	-	37.6 (28.6, 49.5)	52.6 (43.5, 63.7)	-
Time until CMV DNAemia, days, median (IQI)		84 (53, 203)	66 (48, 110)	239 (175, 323)	53 (31, 175)	<0.001	68 (51, 101)	61 (42, 125)	0.3	240 (178, 336)	238 (196, 310)	0.9
Recurrent CMV DNAemia rate at 12 mo***		20.4 (18.5–22.8)	22.1 (19.8–24.9)	19.5 (15.2–25.4)	3.3 (3.3–3.3)	0.002	29.1 (24.7–34.8)	29.1 (24.7–34.8)	-	16.5 (10.8–26.2)	22.1 (16.1–31.6)	-
Clinically significant CMV DNAemia		Censored	182 (25.8%)	76 (38.0%)	<3	<0.001	89 (21.4%)	76 (33.9%)	<0.001	33 (38.8%)	36 (37.9%)	0.9
High CMV DNAemia during follow-up		114 (11.8%)	67 (9.5%)	47 (23.5%)	0 (0.0%)	<0.001	23 (5.5%)	39 (17.4%)	<0.001	17 (20.0%)	25 (26.3%)	0.3
BK polyomavirus infection rate at 12 mo*		11.0 (9.2, 13.1)	10.2 (8.2, 12.7)	13.0 (9.1, 18.6)	13.3 (7.0, 25.4)	0.45	10.4 (7.8, 13.8)	7.6 (4.8, 12.0)	0.1	10.6 (5.7, 19.6)	14.7 (9.1, 23.9)	0.8
Acute rejection, any time during follow-up		231 (23.9%)	181 (25.6%)	32 (16.0%)	18 (30.0%)	0.01	99 (23.9%)	64 (28.6%)	0.2	13 (15.3%)	16 (16.8%)	0.8
Time until (first) acute rejection, days, median (IQI)		23 (9, 214)	22 (9, 175)	122 (10, 400)	15 (9, 61)	​	17 (8, 213)	30 (12, 208)	0.13	318 (32, 409)	70 (9, 411)	0.03
​	Missing	735 (76.1%)	525 (74.4%)	168 (84.0%)	42 (70.0%)	0.11	316 (76.1%)	160 (71.4%)	​	72 (84.7%)	79 (83.2%)	​
Acute rejection type	No acute rejection	735 (76.1%)	525 (74.4%)	168 (84.0%)	42 (70.0%)	​	316 (76.1%)	160 (71.4%)	0.14	72 (84.7%)	79 (83.2%)	0.07
	AMR	19 (2.0%)	16 (2.3%)	3 (1.5%)	0 (0.0%)	0.021	7 (1.7%)	9 (4.0%)		<3	<3	
	TCMR	212 (21.9%)	165 (23.4%)	29 (14.5%)	18 (30.0%)	​	92 (22.2%)	55 (24.6%)		Censored	Censored	
Graft survival rate at 5 years post-KT**		84.7 (81.9, 87.1)	85.2 (82.0, 87.9)	81.0 (74.1, 86.3)	91.1 (77.7, 96.6)	-	90.7 (86.8, 93.5)	89.4 (83.8, 93.1)	-	84.6 (73.7, 91.2)	87.3 (77.5, 93.0)	-
Overall survival rate at 5 years post-KT**		87.7 (85.1, 89.9)	88.1 (85.1, 90.6)	84.6 (78.0, 89.4)	93.6 (81.2, 97.9)	-	92.8 (89.1, 95.2)	90.1 (84.7, 93.7)	-	89.7 (80.4, 94.8)	87.0 (76.3, 93.0)	-
Valganciclovir (any time post-KT)		681 (70.5%)	461 (65.3%)	>197 (>98.5%)	21 (35.0%)	<0.001	234 (56.4%)	185 (82.6%)	<0.001	85 (100.0%)	95 (100.0%)	​
Valganciclovir, timing of first record	At KT (within 30 days)	402 (41.6%)	198 (28.0%)	190 (95.0%)	14 (23.3%)	<0.001	95 (22.9%)	81 (36.2%)	<0.001	79 (92.9%)	92 (96.8%)	0.3
	Later during follow-up	Censored	263 (37.3%)	Censored	7 (11.7%)		139 (33.5%)	104 (46.4%)		6 (7.1%)	3 (3.2%)	
	No treatment	Censored	245 (34.7%)	<3	39 (65.0%)		181 (43.6%)	39 (17.4%)		​	​	

Abbreviations: AMR: antibody-mediated rejection, ATG: anti-thymocyte globulin, CMV: cytomegalovirus, D: donor, IQI: inter-quartile interval, KT: kidney transplantation, PTLN: post-transplant leukopenia or neutropenia, R: recipient, TCMR: T-cell–mediated rejection. *Cumulative incidence, estimate in % (95% CI), **Kaplan-Meier, estimate in % (95% CI), ***Multistate model, estimate in % (95% CI).

### PTLN in KTRs

At 12 months post-KT, 34.5% of patients had experienced at least one PTLN event (Table 2). The incidence was highest among the D+/R-patients (51.5% vs. 31.7% in R+ and 10.0% in D-/R-patients) (Figure 2A; Table 2). In a multivariable logistic regression model adjusting for covariates, the odds of PTLN within 18 months post-KT were 2.42 (p < 0.001) and 0.31 (p = 0.007) for D+/R─ and D─/R─ patients, respectively, with R+ patients as the reference group (Figure 3). Similarly, the incidence of neutropenia was highest among the D+/R─ patients (24.0% vs. 13.6% in R+ patients, p < 0.001) (Figure 2B; Table 2). The proportion of KTRs with PTLN increased during the study period from 3.6% in 2014–2015 to 8.6% in 2020–2022 (Table 2).

**FIGURE 2:**
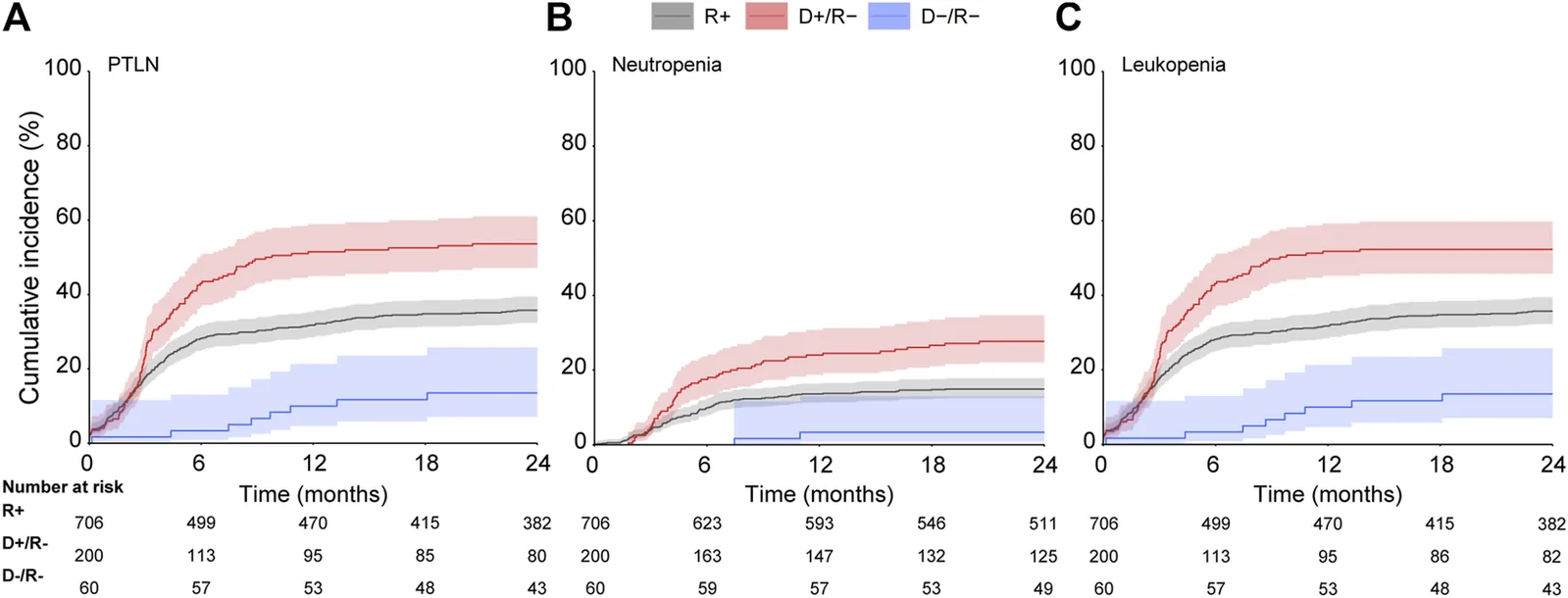
Post-transplant leukopenia/neutropenia. The cumulative incidence of **(A)** PTLN, **(B)** neutropenia and **(C)** leukopenia in R+, D+/R- and D-/R- KTR subgroups. The number of patients at risk at each time point is presented.

**FIGURE 3:**
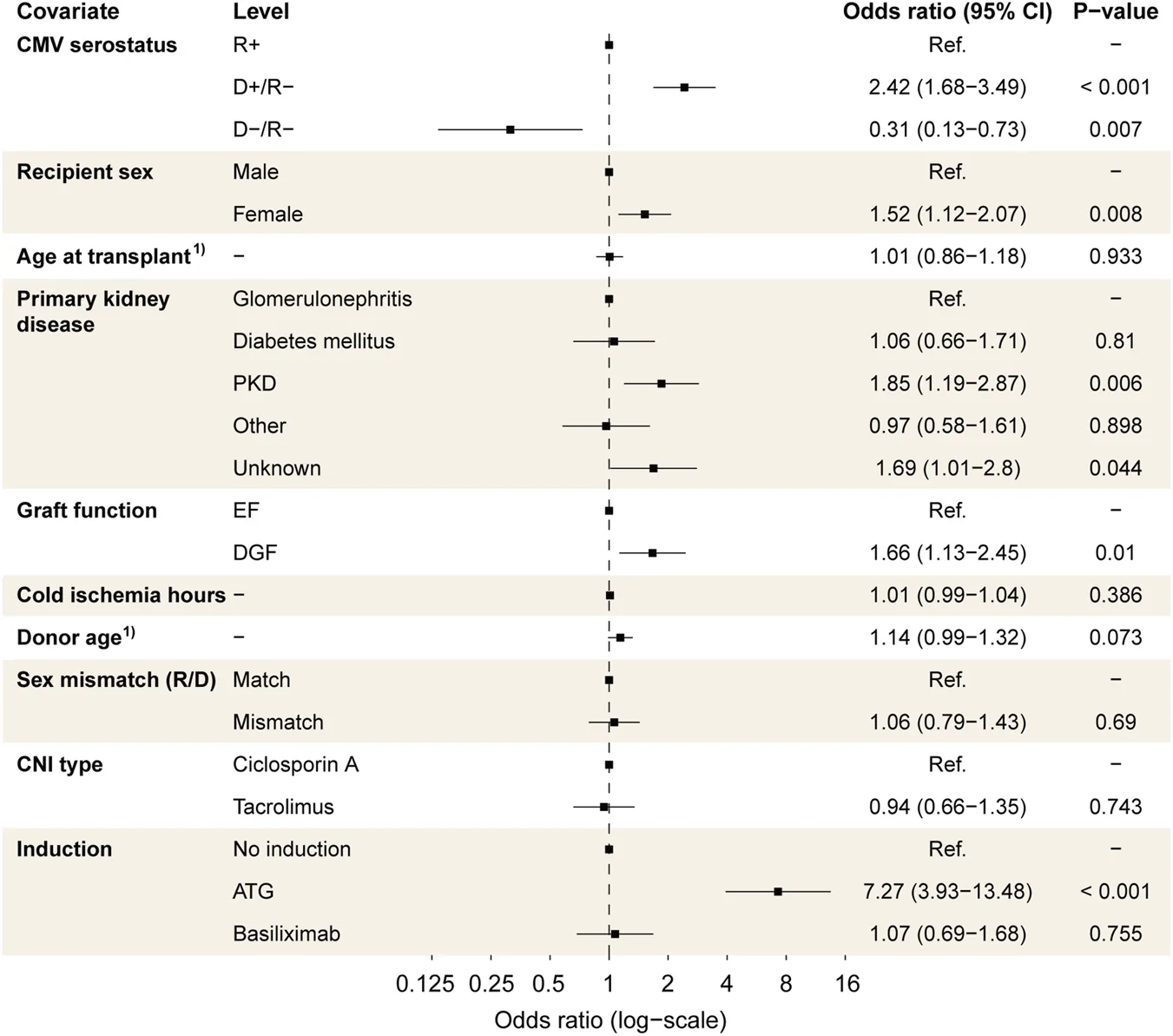
Multivariable logistic regression for PTLN* within 18 months of kidney transplant. *Positive coefficient estimate (or odds above 100%) indicates that the covariate value is positively correlated with the occurrence of PTLN. ^1)^ Odds ratio per 10 years. Abbreviations: ATG: anti-thymocyte globulin, CMV: cytomegalovirus, CNI: calcineurin inhibitor, D: donor, DGF: delayed graft function, EF: early function, PKD: polycystic kidney disease, PTLN: post-transplant leukopenia or neutropenia, R: recipient. ^1^ ICD10: N00, N01, N03-N08, ^2^ ICD10: E10, E11, N08.32, ^3^ ICD10: Q61, excl. Q61.5, ^4^ ICD10: N18.

The incidence of PTLN was associated with female sex (proportion of females vs. males developing PTLN 18 months post KT among D+/R─: 60% vs. 50% and among R+: 41% vs. 31%) and higher CCI score (Supplementary Table S1). Also ATG induction therapy was associated with the risk of PTLN. Over 98% of D+/R-patients received valganciclovir at some point during follow-up and valganciclovir use was associated with the risk of developing PTLN among R+ patients (Table 2). PRA levels were not significantly different between patients with leukopenia and those without leukopenia (Supplementary Table S1).

The proportion of patients with 3 or more PTLN episodes was 9.0% in D+/R─, 5.9% in R+, and 0% in D─/R─ patient groups at 12 months (Supplementary Figures S1A–C; Supplementary Table S2). The corresponding numbers at 24 months were 15.2%, 8.7%, and 0%, respectively. 21.0% of the D+/R─ and 11.8% of all KTRs received G-CSF during the follow-up (Supplementary Table S3).

In a multivariable logistic regression model for PTLN, D+/R─ CMV serostatus, female sex of the recipient, polycystic kidney disease (PKD) as the primary kidney disease, DGF and induction treatment with ATG, were all associated with higher rate of PTLN (P < 0.05, Figure 3).

### CMV DNAemias in KTRs

At 12 months, 45.0% of D+/R─, 47.2% of R+, and 6.7% of D─/R-patients had been observed with at least one episode of CMV DNAemia (Figure 4A; Table 2; Supplementary Table S4). A higher incidence of CMV DNAemias was observed in patients who developed PTLN compared to those that did not (Figures 4B,C; Supplementary Table S4). Clinically significant CMV DNAemia was seen in 38.0% and 25.8%, and high CMV DNAemia in 23.5% and 9.5% of the D+/R─ and R+ KTRs, respectively (Table 2). In R+ KTRs, both clinically significant (33.9% vs. 21.4%, p < 0.001) and high CMV DNAemia was seen more often in patients with PTLN compared to those without (17.4% vs. 5.5%, p < 0.001) (Table 2). In D+/R─ KTRs, there were no differences in the frequencies of clinically significant (37.9% vs. 38.8%, p = 0.9) or high CMV DNAemias (26.3% vs. 20.0%, p = 0.3) by the occurrence of PTLN (Table 2). In D+/R─ patients, most of the CMV DNAemias occurred 6 months after the KT (Figure 4C) while in R+ patients the majority of CMV DNAemias occurred within the first 6 months of the KT (Figure 4B). In R+ patients, 37.3% started valganciclovir later during the follow-up (Table 2).

**FIGURE 4:**
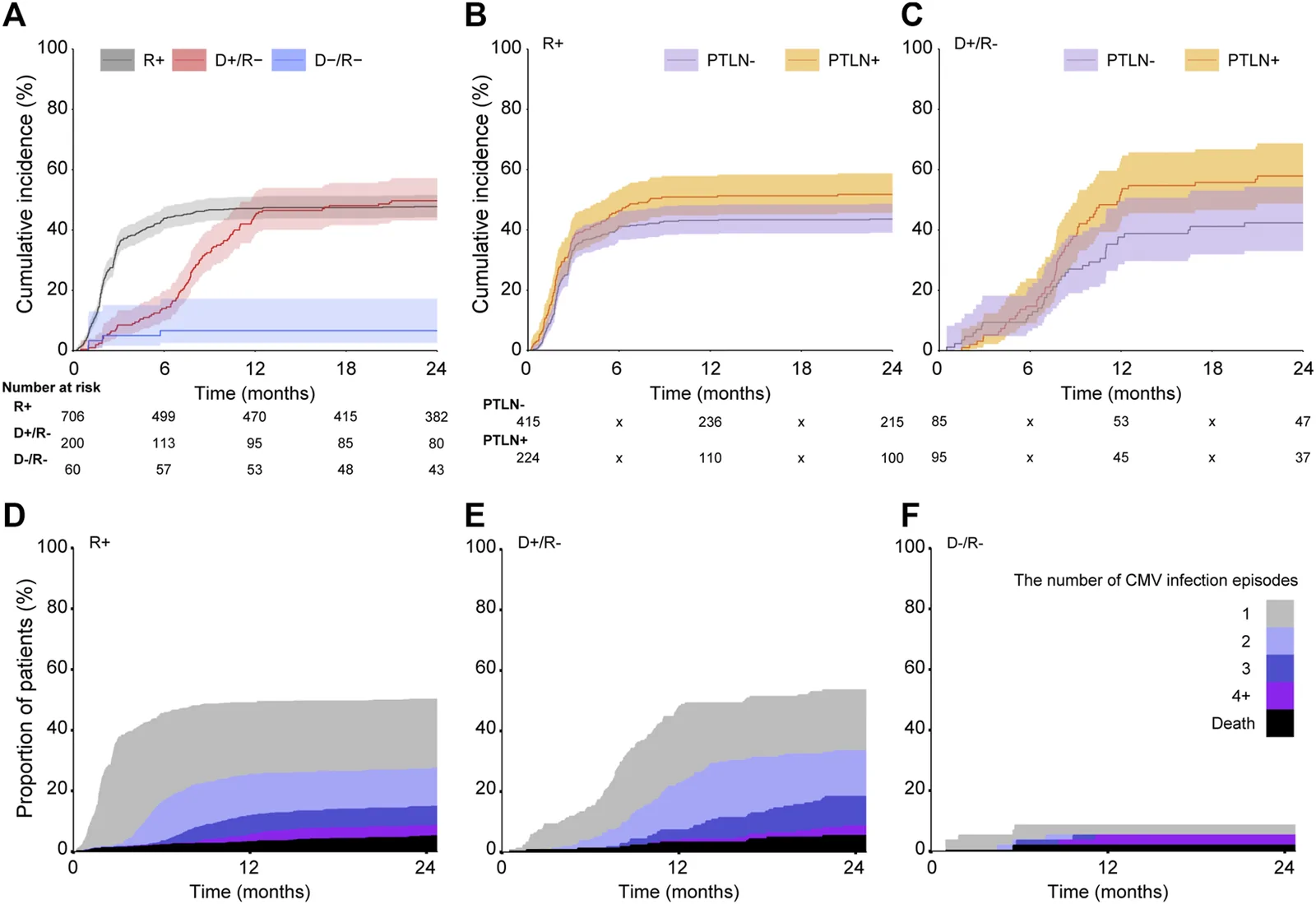
CMV DNAemias in KTRs. The cumulative incidence of the 1st CMV DNAemia in **(A)** in R+, D+/R-, and D-/R- KTR subgroups, and according to PTLN status in **(B)** R+ and **(C)** D+/R- KTRs. A multistate model of recurrent CMV DNAemias in **(D)** R+, **(E)** D+/R-, and **(F)** D-/R- KTR subgroups. See also Supplementary Tables S4, S5.

### Rate and relative timing of CMV DNAemias and PTLN

The rate and timing of the recurrent PTLNs and CMV DNAemias were analyzed by utilizing a multistate model (Supplementary Figure S2A). The incidence of recurrent CMV DNAemias was highest among the D+/R-patients. While at 12 months, the proportion of patients with 2 or more CMV DNAemias was 19.5% in D+/R-, and 22.1% in R+ patients, at 24 months the corresponding numbers were 28.1% and 22.1%, respectively (Figures 4D–F; Table 2; Supplementary Table S5).

In D+/R─patients, a higher rate of PTLN events preceding an episode of CMV DNAemia was observed compared to R+ patients. By the 6-month time point, 34.5% of D+/R─ had developed PTLN, 6.0% CMV DNAemia and 7.5% both PTLN and CMV DNAemia, while among the R+ patients the corresponding numbers were 14.4%, 31.3% and 11.9%, respectively (Figures 5A–C; Supplementary Figure S2B; Supplementary Table S6).

**FIGURE 5:**
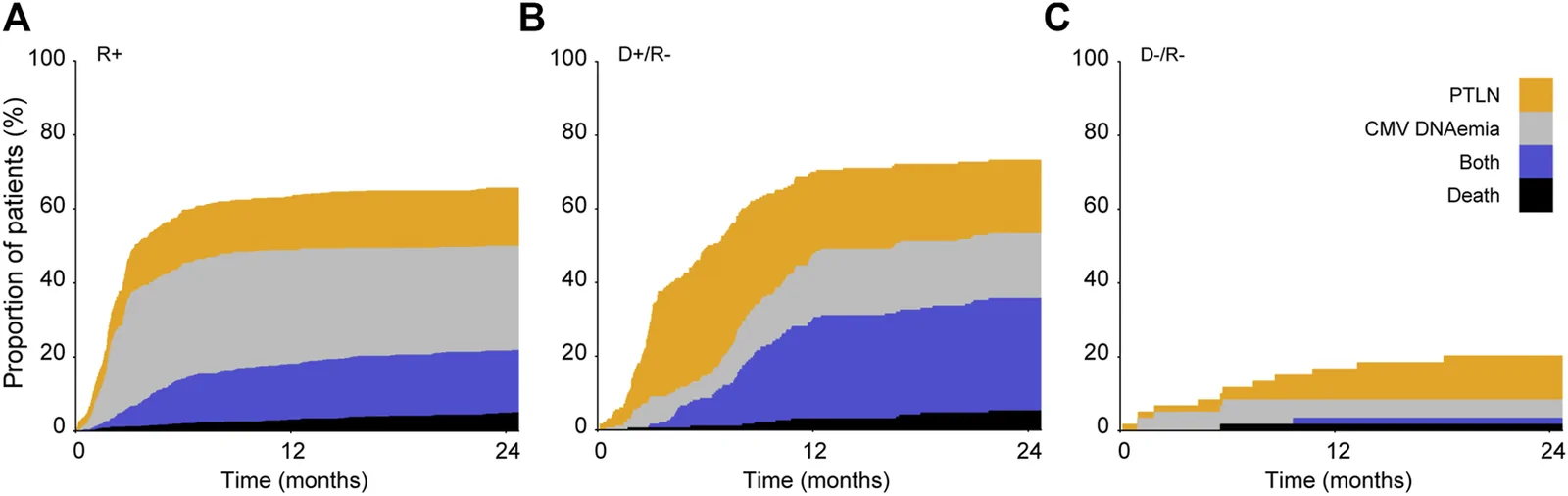
The timing of PTLN and CMV DNAemia. A multistate model of the timing of PTLN and CMV DNAemia in **(A)** R+, **(B)** D+/R, and **(C)** D-/R- KTR subgroups. See also Supplementary Table S6.

### Patient and graft survival

The 5-year survival estimate for all patients in the cohort was 87.7%, and the 5-year graft survival estimate for all patients in the cohort was 84.7% (Supplementary Figures S3A, 4A). No significant differences in patient or graft survival were observed between the subgroups according to CMV serostatus or PTLN status (Supplementary Figures S3B–D, 4B–D). The multivariable Cox models for patient and graft survival showed that advanced age and diabetes mellitus as the primary kidney disease, were associated with a higher risk of both mortality and graft loss (HR > 1, p < 0.05, Supplementary Figures S3E, 4E). In addition, the advanced age of a donor was associated with a higher risk of graft loss (Supplementary Figure S4E). Female sex and PKD compared to glomerulonephritis as the primary kidney disease, were associated with better graft survival (HR < 1, p < 0.05) (Supplementary Figure S4E).

### HCRU analyses

The median duration of the hospital stay related to the transplantation procedure was 11 days (IQI: 8–15), and this was significantly longer among patients who developed PTLN (both D+/R─ and R+) at some stage during the 18-month follow-up time (Supplementary Table S3). At 12 months post-KT, hospital readmission rates due to any reason were 76.0% in D+/R─, 68.8% in R+, and 55.0% in D─/R─ patients. PTLN was associated with higher readmission rates both in the D+/R─ subgroup (PTLN+: 78.9% vs. PTLN-: 72.9%) and the R+ group (75.3% vs. 64.6%). Patients with PTLN had higher ICU admission rates, especially in the R+ group (13.8% vs. 6.0%, p = 0.003).

Overall, the number of healthcare contacts and costs were highest among the D+/R─ patients and lowest among the D─/R─ patients (Figure 6A). Contacts and costs were also analyzed separately according to the specialty (surgery, nephrology, and other) and by the type of contact (inpatient vs. outpatient). The first 30 days were excluded to remove the costs related to the surgical procedure. The exact costs are presented in Supplementary Tables S7, S8. The differences in costs between specialties (surgery, nephrology, and other), and reimbursed medication purchases in relation to CMV serostatus were small during the first-year after KT (Figure 6A). During the years 2–4 post-KT, total costs were higher among the D+/R─ patients (inpatient: 19,961 € per patient (PP), 95% CI: 11,135, 28,786; outpatient: 20,857 € PP, 95% CI: 15,214, 26,499) when compared to R+ (inpatient: 12,212 € PP, 95% CI: 9373, 15,052; outpatient: 16,241 € PP, 95% CI: 14,105, 18,377), and D─/R─ patients (inpatient: 7827 € PP, 95% CI: 0, 16,682; outpatient: 12,769 € PP, 95% CI: 9341, 16,196).

**FIGURE 6:**
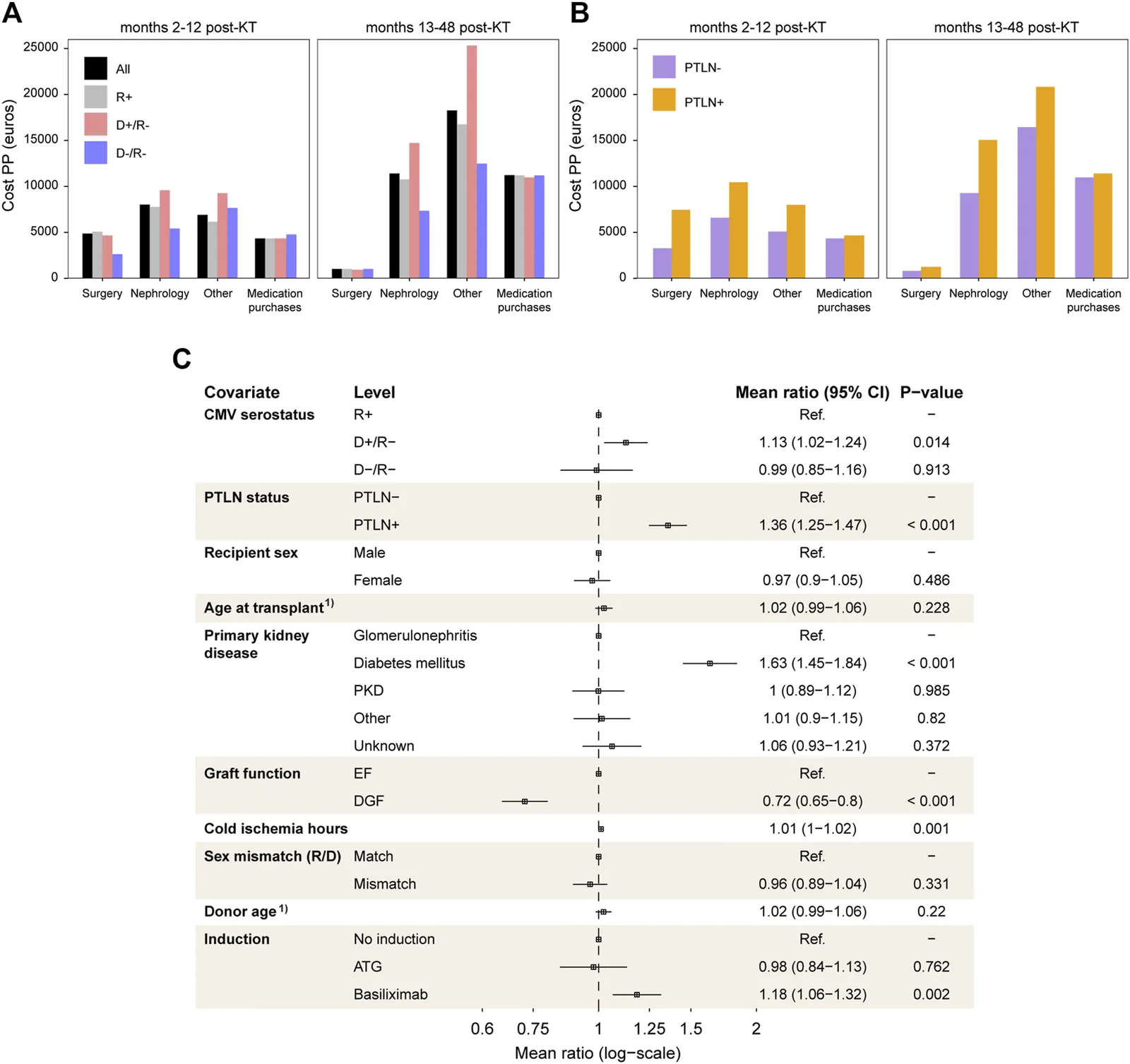
HCRU costs per patient (PP) in euros by **(A)** CMV serotype and **(B)** PTLN status according to the specialty (surgery, nephrology, and other) and reimbursed medication purchases. **(C)** Multivariable Gamma regression model for total HCRU costs including 4 years post-KT. Intercept was 15,567€ per patient year. ^1)^ Mean ratio per 10 years. See also Supplementary Tables S7, S8. Abbreviations: ATG: anti-thymocyte globulin, CMV: cytomegalovirus, D: donor, DGF: delayed graft function, EF: early function, PKD: polycystic kidney disease, PTLN: post-transplant leukopenia or neutropenia, R: recipient.

HCRU was higher in patients with PTLN compared to those without PTLN (Figure 6B; Supplementary Tables S7, S8). During the first-year after KT, the HCRU across all contact types was higher among patients who developed PTLN. The inpatient costs (excluding the first 30 days) for PTLN patients were 14,028 € PP (95% CI: 10,856, 17,201) compared to 5716 € PP (95% CI: 4399, 7033) for patients without an observed PTLN. The corresponding numbers for outpatient costs were 11,695 € PP (95% CI: 10,756, 12,635) and 9045 € PP (95% CI: 8582, 9507), respectively. The costs remained higher among PTLN patients during the second year of follow-up.

In a multivariable gamma regression model for HCRU adjusting for covariates, D+/R─ CMV serostatus, PTLN, diabetes mellitus as the primary kidney disease and induction with basiliximab, were all associated with higher total HCRU (p < 0.05, Figure 6C).

## Discussion

In this study, we investigated the frequency of PTLN and its association with clinical outcomes and HCRU among Finnish KTRs. We found that PTLN is common across all KTRs, with D+/R─ patients having the highest risk. CMV DNAemia occurred at comparable frequencies in R+ and D+/R─ KTRs; however, in D+/R─ patients, the first episode of CMV DNAemia occurred later than in R+ patients. Our analysis using a multistate model suggests that in D+/R─ patients, PTLN tends to precede CMV DNAemia, whereas in R+ patients, CMV DNAemia was more often detected first. We also report that PTLN is associated with increased HCRU and higher healthcare costs in KTRs.

The established time-varying factors associated with the risk of PTLN include induction therapy, antiproliferative and immunosuppressive medication, valganciclovir use for prophylaxis or treatment of CMV, and the use of sulfamethoxazole/trimethoprim [1, 2]. In the present study, ATG was independently associated with a 7-fold increase in the risk of PTLN in the adjusted multivariable logistic regression model. This finding was not unexpected, as prolonged cytopenias are a known side effect of ATG [21]. In addition, ATG induction has been shown to increase the risk of CMV infection [22] and thereby, indirectly, the risk of PTLN in KTRs. The use of ATG has become increasingly common in induction therapy in SOT [23]. In the R+ group, CMV DNAemia was more common in patients with PTLN compared to those without, whereas no such difference was observed in the D+/R─ group. The use of ATG induction therapy was more common in PTLN patients in both groups. This finding is at least partly explained by the immunosuppressive and myelosuppressive effects of ATG and highlights its role as a modifiable risk factor for PTLN. PRA levels did not differ significantly among the R+, D+/R─, and D─/R─ groups, or patients who developed PTLN. We checked the effect of PRA1/2 variables in the multivariable logistic regression models. The hazard ratios were nominal with non-significant p-values and unaffected HRs for other covariates. We could not obtain data on donor specific antibodies (DSA) or detailed individual-level maintenance immunosuppressive therapy, and malignancy history was incorporated only within the CCI. This is a limitation in the multivariable analyses to account for confounding factors.

We found that female sex, PKD and DGF were independently associated with the risk of PTLN. Standard drug-dosing practices may overlook sex-specific differences in drug absorption, distribution and elimination, potentially resulting in unnecessarily high plasma drug peak concentrations, overall drug exposure and increased risk of adverse drug reactions, including PTLN, in women [24–26]. Furthermore, Schellekens et al. reported that patients with autosomal dominant PKD frequently have leukopenia both before and after KT [27]. Although the underlying mechanisms are not fully understood, splenic or hepatic sequestration related to organ enlargement, inflammation-driven alterations in leukocyte turnover, and intrinsic immune dysregulation linked to polycystin dysfunction have been proposed as potential explanations. Although the link between DGF and PTLN has not been described in prior literature, the association was not surprising and may be associated with the overall burden of impaired graft function, comorbidities, perioperative complications, rejections and their treatment. Despite these hypotheses, the relationship between DGF and PTLN remains unclear and warrants further investigation. We observed an increase in PTLN rates from 3.6% in 2014–2015 to 8.6% in 2020–2022. During the study period, some changes occurred in the clinical practice, namely, increasing use of ATG induction, increased use of tacrolimus-based immunosuppression, and moderate increase in the valganciclovir prophylaxis, partly as a results of ABOi transplants and ATG induction. These changes in clinical practice may contribute to the difference.

Although the overall observed CMV DNAemia frequency was comparable between the R+ and D+/R− groups, the multistate analysis provided novel insight into the temporal interplay between CMV DNAemia and PTLN. This model enabled characterization of the sequence in which these events occurred and showed that the pattern differed by CMV serostatus: in R+ recipients, CMV DNAemia more often preceded PTLN, whereas in D+/R− recipients PTLN more frequently occurred first. These findings are clinically plausible considering different valganciclovir use, with shorter 3 months prophylaxis or antiviral treatment after detection of CMV DNAemia more likely in R+ patients and longer 6 months prophylaxis in D+/R− patients. While these observations should be interpreted with caution and do not establish causality, they demonstrate the value of temporal multistate modeling in describing how CMV DNAemia and PTLN evolve over time in routine clinical practice. In this context, the main contribution of the present study lies in providing more nuanced interpretation on the temporal relationships of CMV replication, antiviral exposure and the development of PTLN, while the results are confirmatory for the established clinical associations. Because the retrospective registry-based data did not contain complete information on valganciclovir indications, dosing, or duration, we could not reliably distinguish CMV-driven cytopenia from valganciclovir-induced myelosuppression in R+ recipients. Therefore, the findings should be interpreted with caution in R+ patients. The R+ group was included primarily as a comparator population, whereas the principal clinical focus of the study was the D+/R− group.

Valganciclovir is commonly associated with myelosuppression. Valganciclovir was used as a comparator in the phase 3 study that investigated letermovir in the CMV prophylaxis in D+/R- KTRs [28]. At week 28, 37.0% and 16.5% of the patients receiving valganciclovir had leukopenia and neutropenia , respectively [28]. In another study comparing 100-day and 200-day valganciclovir prophylaxis in D+/R- KTRs, leukopenia was observed in 26% and 38% patients receiving valganciclovir for 100 days or 200 days, respectively, and neutropenia was observed in 15% of the patients in both treatment groups [29]. In that study, the incidence of CMV viremia was lower and the time from the transplantation to CMV viremia was longer in 200-day valganciclovir prophylaxis group. A retrospective study reported that low-dose valganciclovir prophylaxis was associated with a significantly lower incidence of leukopenia compared with standard dosing [30]. The distribution of D+/R─ and R+ patients (18% vs. 82%) was comparable between the groups, and CMV infection occurred in 7% of the patients receiving low-dose and in 9% of those receiving standard-dose valganciclovir prophylaxis. There were more anti-rejection therapy and use of steroids in the standard valganciclovir dose group, which may contribute to the observed higher rate of leukopenia [30]. In a recent prospective observational study, grade 3 or 4 leukopenia or neutropenia was observed in 26.5% of D+/R─ and 7.7% of the D─/R─ KTRs at 6 months [31]. In that study, 91.4% of the D+/R─ patients and 0% of the D─/R─ patients received valganciclovir prophylaxis. In our study, the 6-month leukopenia and neutropenia rates observed in D+/R─ patients were consistent with these findings. Caution is warranted when attributing causality of valganciclovir and leukopenia. Leukopenia is a multifactorial clinical condition with heterogeneous etiologies, in which valganciclovir represents only one of several potential contributing factors, and its specific contribution may be difficult to delineate in the presence of concomitant risk factors. Cutoff values for leukopenia and neutropenia vary between studies having impact on the observed cytopenia rates.

PTLN predisposes patients to opportunistic infections [32], and its management may require dose reduction or discontinuation of immunosuppressive drugs, potentially increasing the risk of graft loss or mortality [33]. Our analysis did not detect a statistically significant association between PTLN and graft loss or mortality, but this may be attributable to the limited number of events. Prior studies investigating the association between PTLN and mortality or acute rejections/graft loss have reported conflicting results [1]. This suggests that the clinical impact of leukopenia may vary across patient populations and that large sample sizes may be required to detect significant associations [1]. Despite different clinical profiles of cyclosporine and tacrolimus, CNI type was not associated with the risk of PTLN. In Finland, mycohenolic acid dose is lower (500 mg b.i.d.) when combined with tacrolimus as compared to the dose (1,000 mg b.i.d) with cyclosporine. This may modulate the risk of PTLN with the type of CNI.

Observational data suggest that pre-emptive KT is associated with improved patient and graft outcomes and reduced morbidity [34], and it may therefore indirectly influence the risk of PTLN. In Finland, pre-emptive KTs were introduced in 2019. Because the number of pre-emptive KTs in this cohort was low, and pre-emptive status did not appear to affect induction therapy or immunosuppression, this variable was not included in the multivariable model. However, residual confounding related to pre-emptive status cannot be excluded.

We also investigated the HCRU and economic impact of PTLN. Our results showed that patients who developed PTLN had higher rates of hospital readmission and ICU admission, and higher overall healthcare costs. The primary HCRU analysis excluded costs from the first 30 days after transplantation to avoid dominance of transplantation procedure-related and perioperative costs, which are common to all KTRs. This design choice improves interpretability of later PTLN-associated HCRU but may underestimate the total economic burden of PTLN, because early leukopenia- or neutropenia-related events, such as monitoring, medication changes, infections, or hospital contacts occurring within the first month, were not captured in the primary cost model. Therefore, the reported cost estimates should be interpreted as conservative estimates of PTLN-associated HCRU after the initial transplant hospitalization period.

The cost analysis should not be interpreted as estimating costs causally attributable to PTLN alone. Many PTLN episodes are managed in the outpatient setting through intensified laboratory monitoring, dose reduction or discontinuation of immunosuppressive and antiviral drugs, which do not generate inpatient costs. In our study, PTLN was defined using laboratory values and the HCRU analysis quantified healthcare contacts and costs associated with PTLN status in routine care. PTLN may be a marker of increased clinical complexity and healthcare resource use rather than as the sole direct cause of the observed cost difference.

While prior studies have suggested increased HCRU among patients with PTLN, most have been conducted in relatively small or narrowly defined patient groups, and often in the context of CMV prophylaxis [31, 35, 36]. Our study provides a broader and more comprehensive evaluation of the economic burden of PTLN in a real-world transplant population, thereby extending existing evidence. These results suggest that PTLN is not only a clinically relevant complication but also associated with healthcare cost formation in kidney transplantation. In the context of an increasingly older and frailer transplant population, the cumulative immunosuppressive burden arising from multiple concomitant therapies may further elevate the risk of PTLN, reinforcing the importance of strategies aimed at mitigating both its clinical and economic consequences.

In this study the information or selection biases were believed to be limited, as all data were obtained from historical registry entries and medical records from a system of universal public healthcare, meaning that the data availability did not depend on access or patient outcomes or clinical status. However, this study is subject to inherent limitations of registry-based observational studies. The data reflects the everyday clinical coding practices and may therefore be non-standardized, incomplete or subject to missing values and variability. For example, due to inconsistent recording of medication dosing between the data sources we could not analyze the dose changes of concomitant medications, including immunosuppressives, or initiations of novel medications before or after detection of PTLN. In addition, DSA status and detailed history of malignancies were not available. Therefore, residual confounding factors remain possible, and these limitations should be considered when interpreting the associations observed in the multivariable models. A further limitation is potential surveillance bias in CMV DNAemia ascertainment. CMV surveillance was not protocol-standardized and likely differed between patient groups. Consequently, CMV DNAemia frequencies should be interpreted as comparisons of detected CMV DNAemia in routine care, not necessarily true underlying incidence. Our findings are generalizable to the entire population of Finland but may not be applicable as such in other countries due to differences in clinical and healthcare practices.

In conclusion, PTLN was common among KTRs in Finland, particularly in D+/R− recipients, and its clinical risk factors were largely consistent with previous literature. The main contribution of this study is the demonstration that PTLN is associated with increased healthcare resource utilization and higher costs in a real-world Finnish transplant population, and the use of multistate modeling to characterize the temporal relationship between PTLN and CMV DNAemia. These findings support the need to consider both clinical and economic consequences when evaluating approaches to reduce myelosuppressive complications after kidney transplantation.

## Data Availability

The data analyzed in this study is subject to the following licenses/restrictions: this study was based on the secondary use of healthcare register data. Only the registry personnel had full access to patient data. The single-level data cannot be shared. Only the registry holders have the authority to grant rights to third parties for data usage in accordance with the Act on Secondary Use of Health and Social Data 552/2019, Finland. Requests to access these datasets should be directed to Findata: info@findata.fi.
