# Supplementary material for: The frequency and timing of leukopenia and CMV infections in kidney transplant recipients in Finland – a retrospective observational study
Source: Transpl Int. 2026 Oct 1;39:16893. doi: 10.3389/ti.2026.16893 (PMC13668761; doi:10.3389/ti.2026.16893)
Supplement: Supplementary file 1 [file Supplementaryfile1.docx]

**Supplementary material**

**Supplementary methods**

PTLN status for the primary analyses was defined based on the first PTLN episode occurring within 18 months after kidney transplantation. This predefined window was used to focus on the early post-transplant period, during which CMV prophylaxis, CMV DNAemia monitoring, immunosuppressive treatment changes, and infection-related complications are most relevant. Following this the outcome analyses stratified by the PTLN status were done by applying landmark analysis i.e., only patients whose follow-up extended to at least 18 months in order to mitigate immortality survival bias from not having enough follow-up time for some patients to observe PTLN.

CMV DNAemia was identified from recorded peripheral blood plasma CMV nucleic acid test results. In routine Finnish clinical practice during the study period, CMV monitoring differed according to CMV risk group and clinical context. D+/R− recipients routinely received 6 months of valganciclovir prophylaxis and were monitored as part of post-transplant follow-up. R+ recipients were monitored after transplantation or received 3 months valganciclovir prophylaxis when clinically indicated, including in association with suspected CMV-related symptoms or higher-risk clinical situations such as ABO-incompatible transplantation, lymphocyte-depleting induction therapy, or treatment of acute rejection. CMV DNAemia was not routinely monitored during prophylaxis. In patients not receiving prophylaxis, or after prophylaxis ended, CMV DNAemia was monitored with 2-6 weeks intervals based on clinician’s discretion and local protocols. Because this retrospective registry-based data did not include complete structured information on the indication or planned schedule for each CMV test, CMV DNAemia outcomes represent detected CMV DNAemia in routine care rather than surveillance protocol-driven incidence estimates.

Detailed individual- level data on immunosuppressive drug doses and trough levels were not available for the purposes of this study. Immunosuppression was a combination of cyclosporine or tacrolimus, mycophenolate (500mg b.i.d with tacrolimus and 1000mg b.i.d with cyclosporine), and steroids. Induction with basiliximab or anti-thymocyte globulin was given to patients with previous sensitization or higher number of HLA mismatches. Trough level targets for tacrolimus were 7-10 ng /ml for the first three months, and 170-200 for cyclosporine, decreasing thereafter. Steroids were slowly withdrawn in most patients during the first post-transplant year.

**Supplementary Figures**

**Supplementary Figure S1. Recurrent PTLNs**

A multistate model of recurrent PTLNs in **(A)** R+, **(B)** D+/R-, and **(C)** D-/R- KTR subgroups. See also Supplementary Table S2.

**Supplementary Figure S2. Multistate models**

The multistate model designs for **(A)** recurrent CMV DNAemias and **(B)** relative timing of PTLN and CMV DNAemias.

**Supplementary Figure S3. Patient survival**

Kaplan-Meier overall survival (OS) curves of **(A)** all KTRs and in **(B)** R+, D+/R-, and D-/R- KTR subgroups, and according to PTLN status in **(C)** R+ and **(D)** D+/R- KTRs. 5-year survival estimates with 95% CIs are presented. **(E)** Multivariable Cox regression model for patient survival.

**Supplementary Figure S4. Graft survival**

Kaplan-Meier graft survival curves of **(A)** all KTRs and in **(B)** R+, D+/R-, and D-/R- KTR subgroups, and according to PTLN status in **(C)** R+ and **(D)** D+/R- KTRs. 5-year graft survival estimates with 95% CIs are presented. **(E)** Multivariable Cox regression model for graft survival.

**Supplementary tables**

Please see a separate excel file for the Supplementary tables.

**Supplementary table S1. Characteristics**

**Supplementary table S2. Recurrent PTLNs**

**Supplementary table S3. Medications and hospitalization**

**Supplementary table S4. CMV DNAemias**

**Supplementary table S5. Recurrent CMV DNAemias**

**Supplementary table S6. CMV DNAemia and PTLN timing**

**Supplementary table S7. HCRU by type**

**Supplementary table S8. HCRU, total costs**

Numbers are per patient cost estimates (95% CISs) for the corresponding period.
